# Humanin Treatment Protects Against Venetoclax-Induced Bone Growth Retardation in *Ex Vivo* Cultured Rat Bones

**DOI:** 10.1210/jendso/bvae009

**Published:** 2024-01-25

**Authors:** Lilly Velentza, Malin Wickström, Per Kogner, Claes Ohlsson, Farasat Zaman, Lars Sävendahl

**Affiliations:** Division of Pediatric Endocrinology, Department of Women's and Children's Health, Karolinska Institutet, 171 65 Stockholm, Sweden; Division of Pediatric Oncology and Surgery, Department of Women's and Children's Health, Karolinska Institutet, 171 65 Stockholm, Sweden; Division of Pediatric Oncology and Surgery, Department of Women's and Children's Health, Karolinska Institutet, 171 65 Stockholm, Sweden; Astrid Lindgren Children's Hospital, Karolinska University Hospital, 171 64 Stockholm, Sweden; Centre for Bone and Arthritis Research, Department of Internal Medicine and Clinical Nutrition, Institute of Medicine, Sahlgrenska Academy, University of Gothenburg, 413 45 Gothenburg, Sweden; Division of Pediatric Endocrinology, Department of Women's and Children's Health, Karolinska Institutet, 171 65 Stockholm, Sweden; Division of Pediatric Endocrinology, Department of Women's and Children's Health, Karolinska Institutet, 171 65 Stockholm, Sweden; Astrid Lindgren Children's Hospital, Karolinska University Hospital, 171 64 Stockholm, Sweden

**Keywords:** venetoclax, humanin, growth, metatarsals, neuroblastoma

## Abstract

**Context:**

Recent preclinical studies reported that the BCL-2 inhibitor venetoclax can impair bone growth. A strategy to prevent such a side effect of this promising anticancer drug is highly desired. Earlier *in vitro* and *in vivo* studies suggested that the mitochondrial peptide humanin has the potential to prevent drug-induced growth impairment.

**Objective:**

We hypothesized that co-treatment with the humanin analog HNG may prevent venetoclax-induced bone growth impairment.

**Methods:**

*Ex vivo* studies were performed in fetal rat metatarsal bones and human growth plate samples cultured for 12 and 2 days, respectively, while *in vivo* studies were performed in young neuroblastoma mice being treated daily for 14 days. The treatment groups included venetoclax, HNG, venetoclax plus HNG, or vehicle. Bone growth was continuously monitored and at the end point, histomorphometric and immunohistochemical analyses were performed in fixed tissues.

**Results:**

Venetoclax suppressed metatarsal bone growth and when combined with HNG, bone growth was rescued and all histological parameters affected by venetoclax monotherapy were normalized. Mechanistic studies showed that HNG downregulated the pro-apoptotic proteins Bax and p53 in cultured metatarsals and human growth plate tissues, respectively. The study in a neuroblastoma mouse model confirmed a growth-suppressive effect of venetoclax treatment. In this short-term *in vivo* study, no significant bone growth-rescuing effect could be verified when testing HNG at a single dose. We conclude that humanin dose-dependently protects *ex vivo* cultured metatarsal bones from venetoclax-induced bone growth impairment by restoring the growth plate microstructure.

##  

Treatment-associated adverse effects are common in childhood cancer patients and long-term survivors who experience chronic health conditions many years after the cessation of therapy [1, 2]. Among these complications, skeletal fragility and suppression of bone growth arise frequently in growing children treated for cancer and this may lead to permanent bone health impairment, pathological fractures, and chronic pain [3-5].

Increasing evidence supports that various anticancer therapeutic modalities have a negative effect on longitudinal bone growth, either indirectly via hormonal disturbances (ie, growth hormone, sex hormones, thyroid hormones) [6] or directly by dysregulating the function of chondrocytes in the growth plate [7], the cartilaginous structure where longitudinal bone growth takes place [8]. Among the different drug classes, both chemotherapeutic agents (eg, glucocorticoids, alkylators) and newer selective inhibitors (eg, proteasome and hedgehog inhibitors) have been shown to cause cellular damage locally in the growth plate cartilage, resulting in irreversible bone growth suppression [9-13]. We have recently shown that venetoclax, a promising selective BCL-2 inhibitor, induced growth retardation in *ex vivo* and *in vivo* experimental models [14].

Despite the progress in the efforts being undertaken for rigorous investigation and early identification of treatment-induced skeletal toxicities in childhood cancer, the available therapeutic or preventive strategies to protect bone tissue remain scarce. In the longitudinal bone growth setting, growth hormone is the only agent that is currently used in selected cases but concerns regarding long-term safety remain to be resolved [15]. Thus, there is a great need for new approaches that will improve bone health during and after childhood cancer treatment, without hindering the antitumor efficacy of the administered regimens.

An opening for the possible prevention of growth impairment caused by anticancer drugs arrived when the synthetic mitochondrial peptide HNG was reported to rescue from bortezomib and glucocorticoid-induced bone growth suppression when tested in different preclinical models [16, 17]. HNG belongs to a larger category of synthetic peptides constructed after the discovery of humanin, an endogenously produced mitochondrial peptide that has been initially shown to decrease neuronal cell death in Alzheimer's disease [18]. Numerous preclinical studies have demonstrated that humanin/HNG exert anti-apoptotic, anti-inflammatory, and antioxidative effects in various tissues and conditions, including neurodegenerative diseases, hypogonadism, myocardial ischemia, and gestational diabetes mellitus [19]. In addition, recent studies have highlighted a central role of humanin in the lifespan by showing that humanin levels decreased with age in various species and that humanin overexpression extended the lifespan of *C elegans* [20]. From a mechanistic perspective, accumulating data show that humanin/HNG regulate major cellular processes by interacting with various molecules and consequently controlling signaling cascades including, but not limited to, insulin/IGF-I, JAK2/STAT3, PI3K/Akt, and ERK1/2 [21-25].

Based on the reported cytoprotective properties of HNG, we hypothesized that the humanin analog HNG has the potential to prevent bone growth impairment caused by venetoclax monotherapy. Preclinical studies were performed in cultured rat metatarsal bones, cultured human growth plate tissue samples and, as venetoclax is under clinical development for the treatment of neuroblastoma, also in an *in vivo* neuroblastoma xenograft mouse model.

## Materials and Methods

### Compound Preparation

Venetoclax (MedChemExpress) was diluted in dimethyl sulfoxide for the *in vitro* and *ex vivo* experiments and stock solutions were stored at −20 °C. For the *in vivo* experiments, venetoclax was formulated in 60% PHOSAL 50 PG, 30% polyethylene glycol 400 (PEG 400), and 10% ethanol and was prepared fresh on the day of treatment. The humanin analog HNG (Met-Ala-Pro-Arg-Gly-Phe-Ser-Cys-Leu-Leu-Leu-Leu-Thr-Gly-Glu-Ile-Asp-Leu-Pro-Val-Lys-Arg-Arg-Ala) was purchased from GenScript and was dissolved in sterile saline for *ex vivo* and *in vivo* use.

### *Ex Vivo* Culture of Fetal Rat Metatarsal Bones

E13 Sprague-Dawley pregnant rats were purchased from Janvier Labs and allowed to acclimatize in the animal facility for 7 days. Water and standard food pellets were provided *ad libitum*. Thereafter, the rat dams were humanely euthanized via CO_2_ on day 20 of gestation and the E20 embryos were obtained. After decapitation, the hind paws were collected and the 3 middle metatarsal bones were microdissected under an inverted microscope, as previously described [26]. Next, the metatarsals were transferred to 24-well plates and randomly distributed to the following groups: (i) control; (ii) venetoclax 3.75 μM; (iii) HNG 10, 20, or 40 μM; and (iv) the combination of venetoclax and HNG. The culture medium used was Dulbecco’s modified Eagle’s medium (DMEM)/F12 (Gibco) supplemented with 50 μg/mL ascorbic acid (Sigma-Aldrich), 1 mM sodium glycerophosphate (Sigma-Aldrich), 0.2% bovine serum albumin (Sigma-Aldrich), and 20 μg/mL gentamicin, and the compounds of interest (venetoclax, HNG) were diluted in the medium from stock solutions. Medium changes together with image capture (Hamamatsu C4742-95 digital camera mounted on a Nikon SMZ-U microscope) and length measurements (Infinity Analyze software, Lumenera Corporation) were performed on days 0, 2, 5, 7, 9, and 12 (termination). Three independent experiments were performed, and each metatarsal bone was considered an experimental unit.

### *Ex Vivo* Culture of Human Growth Plate Cartilage

Growth plate biopsies were collected from the proximal femur and distal tibia in 1 constitutionally tall 13-year-old mid-pubertal male patient undergoing epiphysiodesis surgery aiming to reduce his further growth. After collection, the biopsies were directly placed in ice-cold medium (DMEM–high-glucose medium without phenol red supplemented with 10 µg/mL gentamicin) and further processed according to our standard protocol [27]. Briefly, human growth plate tissues were cut into small pieces (0.5-1 mm) and individually transferred to 24-well plates containing culture medium (DMEM–high-glucose medium without phenol red supplemented with 10 µg/mL gentamicin, 50 µg/mL ascorbic acid, 1 mM beta-glycerophosphate, and 0.2% bovine serum albumin). Venetoclax and/or HNG were added to the medium, while for the control group dimethyl sulfoxide/saline was added. Cultures were terminated after 48 hours and the tissue pieces were then fixed in 4% paraformaldehyde, decalcified in 10% EDTA, and placed in 70% ethanol until paraffin embedding.

### Neuroblastoma Xenograft Mouse Model

Female NMRI nude mice aged 3.5 to 4 weeks (BomTac: NMRI-*Foxn1^nu^*) were supplied by Taconic Biosciences. After a 7-day adaptation period, mice were injected subcutaneously into the right flank with SK-N-BE (2) neuroblastoma cells (100 μL, 12.5 × 10^6^ cells in culture media without antibiotics or fetal bovine serum) under mild isoflurane anesthesia. Mice were monitored daily for tumor development. When palpable tumors reached a volume of 150 mm^3^, the mice were stratified based on their body weight in one of the following treatment groups: (i) venetoclax (100 mg/kg daily via oral gavage, n = 13) (ii), HNG (100 μg/kg daily, intraperitoneally, n = 12), (iii) combination of venetoclax + HNG (daily, n = 13), or (iv) vehicle solution (control, n = 14). The duration of the treatment was 14 days and the tumors were measured every other day with a digital caliper. Tumor volume was calculated based on the formula: width^2^ × length × 0.44, where width represents the smallest tumor dimension [28]. Tumor volume index was calculated as the measured tumor volume each day divided by the volume on the day of inclusion in the study (d0). The mice were monitored daily for any signs of tumor or treatment-related discomfort and weight measurements were taken every other day. Water and food pellets were given *ad libitum*. Following our pre-established humane end points, one mouse in both the vehicle and the HNG group was humanely culled earlier than the experimental end point due to increased tumor volume (>2.0 mL). No mouse died due to drug-related toxicity or tumor burden. On the first day of treatment (d0) and at the end point (d14), X-rays of the mice were taken under mild isoflurane anesthesia as previously described and the lengths of the right tibia bones were measured in a blinded manner [17]. The results of all mice included in the experiment were analyzed.

### Tissue Preparation and Histomorphometry

At the termination of each experiment, metatarsals from each group (n = 2 to 3) were washed in cold phosphate-buffered saline and fixed in 4% paraformaldehyde for 24 hours. For the groups in which HNG was used, we selected the metatarsals treated with the lowest (10 μμ) and highest (40 μμ) concentration for further analyses. For the *in vivo* studies, the long bones from all mice were collected after euthanasia, fixed in 4% formaldehyde for 48 hours at +4 °C, and decalcified in 10% EDTA for 1 week. Paraffin sections (5 μm) were prepared with the same bone orientation along the longitudinal axis, and bones from all treatment groups were placed on the same slide. For histomorphometric analyses, the sections were deparaffinized, rehydrated, and stained with Alcian blue solution (1% in 3% acetic acid, pH 2.5, Sigma-Aldrich) for 10 minutes and counterstained with Nuclear Fast red solution (Sigma-Aldrich) for 10 minutes. Then, the slides were dehydrated and mounted with Pertex (Histolab) and microscopic images were captured (Leica Microsystems). The total growth plate height in mouse tibias was calculated as an average of 10 measurements taken in the mid–two-thirds of the growth plate area and along the chondrocyte columns. The height of the resting + proliferative (R + P) zone was measured at 10 different regions of the metatarsal growth plate, and the size of hypertrophic cells was averaged based on measurements from 10 different hypertrophic cells at the proximal and distal ends. A cell was considered hypertrophic if the longitudinal height was bigger than 7 μm, as previously described [29]. Analyses were performed in a blinded manner with Image J software (National Institutes of Health).

#### Immunostaining

Immunohistochemistry was performed as previously described [30]. Briefly, sodium citrate buffer (10 mM, pH 6.0) was used for antigen retrieval, which was performed for 15 minutes at 75 °C. Goat serum was used for blocking for 1 hour followed by incubation with the primary antibody overnight at 4 °C. The primary antibodies used in the study were the following: anti-proliferating cell nuclear antigen (PCNA) antibody (1:3000; Abcam catalog No. ab18197, RRID:AB_444313), anti-Bax antibody (1:500; Abcam catalog No. ab32503, RRID:AB_725631), anti-Bcl-2 antibody (1:100; Abcam catalog No. ab196495, RRID:AB_2924862), humanin antibody (1:100; Novus catalog No. NB100-56877, RRID:AB_838379), and p53 antibody (1:200; Santa Cruz Biotechnology catalog No. sc-393031, RRID:AB_3083496). Next, the secondary antibody (1:300-1:500; Abcam catalog No. ab97049, RRID:AB_10679577) was added for 1 hour, followed by incubation with an avidin-peroxidase complex (Vectastain ABC-kit PK-6100) and development with 3,3′ diaminobenzidine (DAB) (Dako K3468). Alcian blue solution was used as counterstain. Photos of the slides were taken with a microscope (Leica Microsystems), and the plugin IHC toolbox in Image J software (National Institutes of Health) was used to quantify the percentage of positive staining in each section where the threshold was kept the same for all groups. For human growth plate tissues, the total number of positive cells per millimeter squared growth plate area was measured. All analyses were performed in a blinded manner.

### Peripheral Quantitative Computed Tomography

Peripheral quantitative computed tomography (pQCT) scans were performed for the analyses of bone parameters using peripheral quantitative computed tomography XCT Research M (software version v.4,5B; Norland Stratec), as previously described [31]. Femurs obtained from neuroblastoma xenograft mice were scanned at the mid-diaphyseal and metaphyseal for cortical and trabecular bone parameters, respectively.

### Gene Expression Analysis

Human chondrogenic USAC cells [32] were seeded in 6-well plates (200 000 cells per well) in DMEM/F12 medium + 10% fetal bovine serum (Gibco) until confluent. Thereafter, cells were treated with venetoclax (2 μμ), HNG (10 μμ), or the combination of the 2 compounds for 24 hours. Total RNA was extracted using the Zymo Quick-RNA microprep kit (Zymo Research) and complementary DNA was synthesized using the iScript cDNA Synthesis Kit (Bio-Rad) as per the manufacturer's protocol. Quantitative reverse-transcription polymerase chain reaction was performed (CFX96 Touch Real-Time PCR Detection System, Bio-Rad) for the following genes: *SOX9*, *ATG7*, and *STAT3* (PrimePCR SYBR Green Assay, Bio-Rad) and the values were normalized to glyceraldehyde-3-phosphate dehydrogenase (*GAPDH*). Gene expression levels were determined using the ΔΔC_T_ method (fold change: 1.0 for the untreated control).

### Ethical Approvals

All animal studies were approved by the local ethics committee (Stockholm North Animal Ethics Committee, permit Nos. 13820-2019 and 13572-2018) and were carried out in accordance with the Directive 2010/63/EU for animal experiments, national regulations, and the ARRIVE guidelines (https://arriveguidelines.org). The local ethics committee approved the collection of human growth plate material (Karolinska Institutet Research Ethics Committee North at the Karolinska University Hospital, Stockholm, Sweden, permit No. 97-214). Informed consent was obtained from each patient and their legal guardians and documented in the original hospital records. All experiments were performed in accordance with the relevant guidelines and regulations.

### Statistical Analyses

All analyses were performed with GraphPad Prism 9.1.1. Mean values ± SDs are presented unless otherwise specified. One-way analysis of variance followed by the Sidak multiple comparison test or Kruskal-Wallis test was applied when comparing multiple groups. *P* values less than .05 were considered statistically significant.

## Results

### Effects of Venetoclax and HNG on the Growth of Cultured Fetal Rat Metatarsal Bones

**Figure 1.**
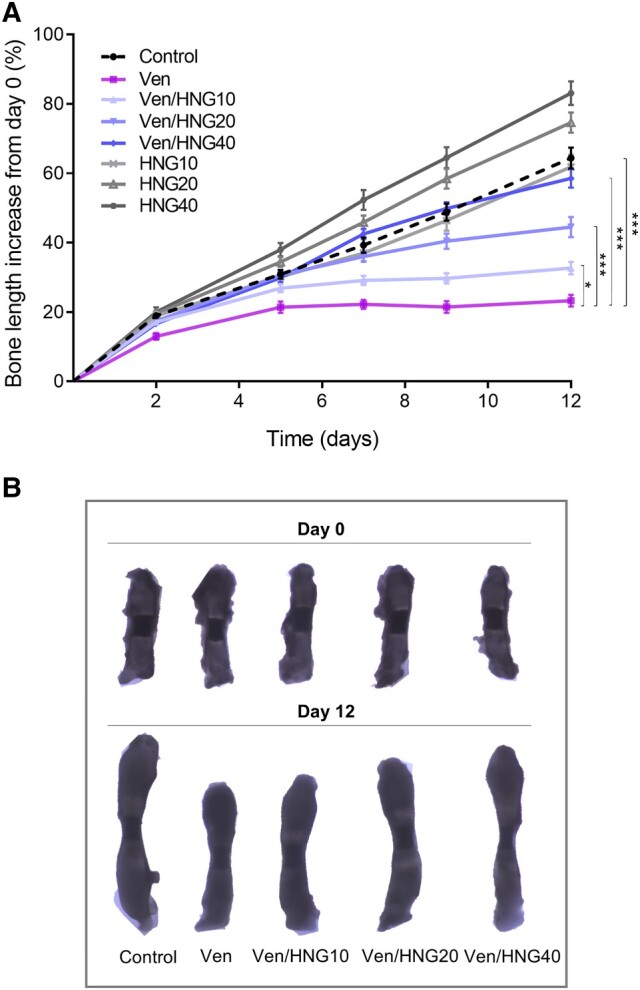
HNG treatment rescued from venetoclax-induced bone growth retardation in *ex vivo* cultured E20 fetal rat metatarsal bones. A, The graph shows growth curves for each treatment group over the 12-day-culture period. The percentage (%) of bone length increase from day 0 was calculated for each time point. Venetoclax (Ven) suppressed bone growth while co-treatment with HNG (10-40 μM) dose-dependently rescued bone growth and at the highest dose of HNG (40 μμ) tested, bone growth was similar to control bones when assessed after 12 days of treatment. Three independent experiments were performed. Data are shown as means ± SEMs, n = 18/group; *P less than .05 and ***P less than .001. B, Representative images of *ex vivo* cultured metatarsal bones captured on day 0 and day 12. For each group, the same metatarsal bone is shown at both time points.

When venetoclax (3.75 μM) was added to the culture medium, fetal rat metatarsal bone growth was markedly suppressed when compared to control bones (Fig. 1A and 1B; 23.3 ± 1.7% vs 64.4 ± 3.0% bone length increase from day 0 to day 12, respectively; *P* < .001). When venetoclax was combined with HNG (10, 20, or 40 μM), a dose-dependent bone growth-rescuing effect was observed (see Fig. 1A and 1B; Ven/HNG10: 32.7 ± 1.8% bone length increase; *P* < .05 vs Ven; Ven/HNG20: 44.5 ± 2.9%; *P* < .001 vs Ven, and Ven/HNG40: 58.5 ± 2.7%; *P* < .001 vs Ven). Interestingly, venetoclax-induced bone growth suppression was almost entirely prevented by the highest concentration of HNG tested (see Fig. 1A and 1B; Ven/HNG40: 58.5 ± 2.7% vs control 64.4 ± 3.0%; *P* = .4).

### Effects of Venetoclax and HNG on Chondrogenesis in Cultured Metatarsal Bones

**Figure 2.**
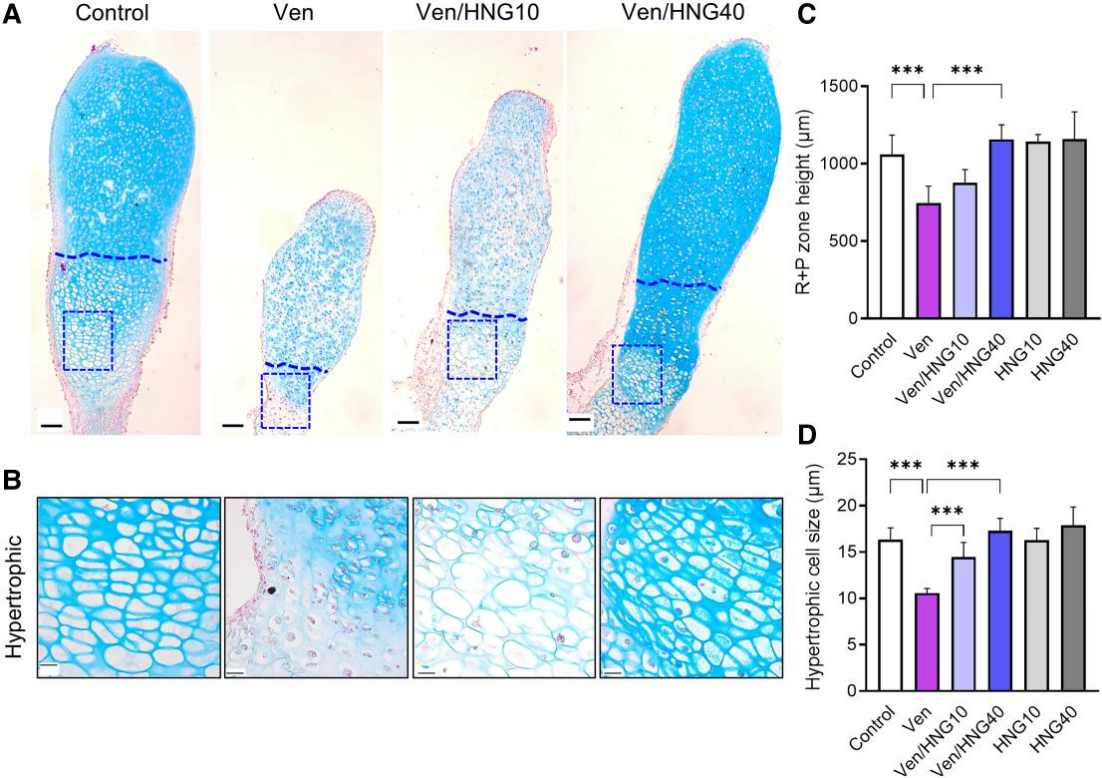
HNG restored metatarsal bone histology when combined with venetoclax. A, Representative images of control, Venetoclax (Ven), and Venetoclax + HNG-treated (Ven/HNG10 and Ven/HNG40) metatarsals stained with Alcian blue and nuclear fast red solution. The box highlights the area of the hypertrophic zone shown in B. Original magnification 5×, scale bar = 100 μm. B, Representative micrographs of the hypertrophic zone in metatarsal bones from each group. Original magnification 40×, scale bar = 20 μm. C and D, Quantification of histomorphometric analyses. Venetoclax reduced the resting + proliferative (R + P) zone height and the hypertrophic cell size. Combination treatment with Ven + HNG significantly improved both these parameters. Data are shown as means ± SDs, n = 4 to 6/group; ***P less than .001.

**Figure 3.**
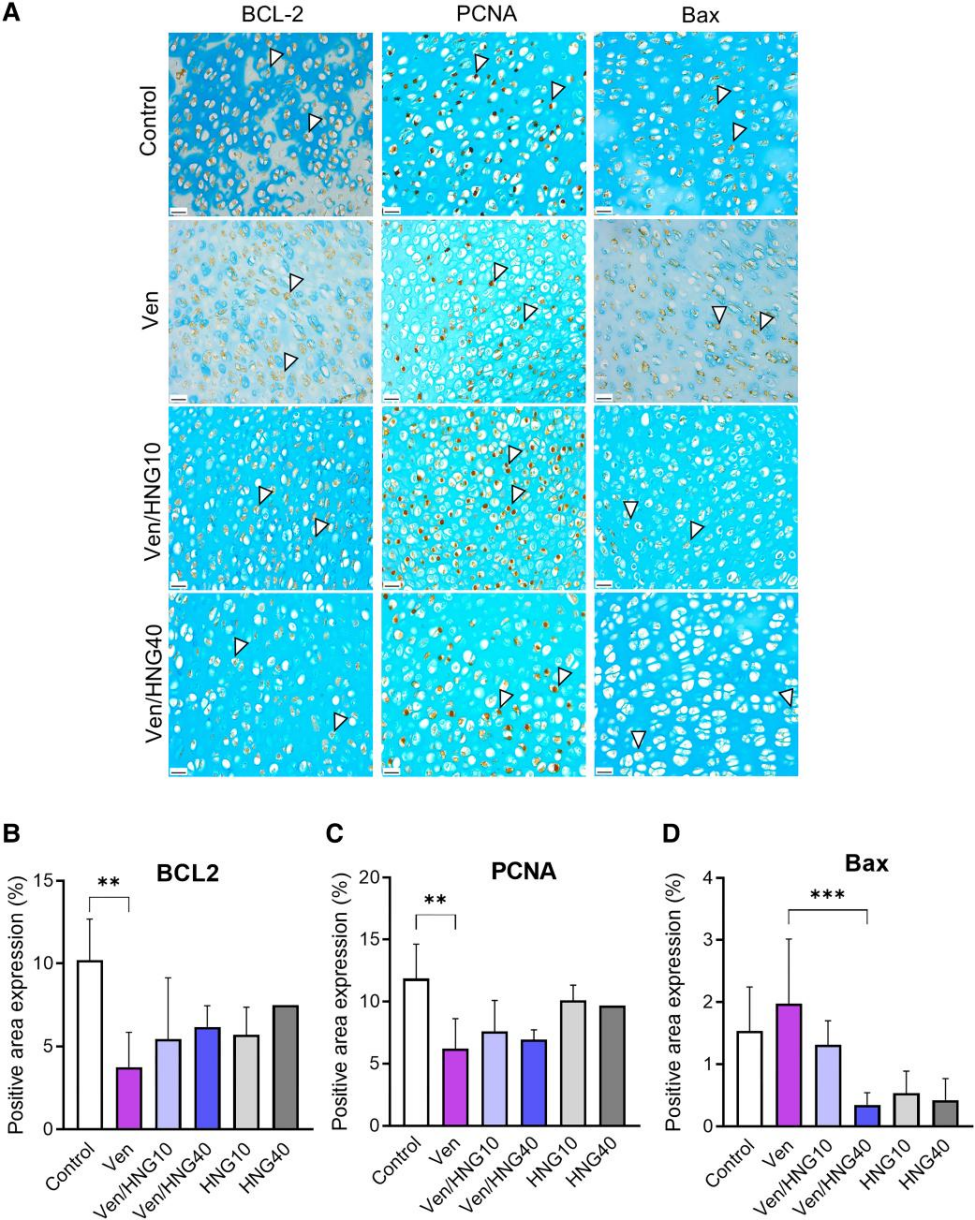
Effects of venetoclax and/or HNG treatment on protein expression in fetal rat metatarsal bones. A, Representative microscopic images of venetoclax (Ven) and/or HNG-treated metatarsals stained for BCL-2, PCNA, and Bax, counterstained with Alcian blue. Original magnification 40×, scale bar = 20 μm. Representative positive cells are marked with arrows. B to D, Quantification of BCL-2, PCNA, and Bax staining in metatarsal bones. Venetoclax significantly decreased the expression of BCL-2 and the proliferative marker PCNA. Combination treatment with Ven + HNG significantly downregulated the proapoptotic marker Bax compared to venetoclax alone. Data are shown as means ± SDs, n = 1 to 8/group; **P less than .01 and ***P less than .001.

Histomorphological analysis of the metatarsals showed that the height of the R + P zone was significantly reduced in venetoclax-treated bones (Fig. 2C; 747 ± 48 μm in Ven vs 1060 ± 56 μm in control; *P* < .001), an effect that was reversed when co-treating with Ven + HNG (see Fig. 2C; 878 ± 35 μm in Ven/HNG10; *P* = NS vs Ven and 1158 ± 38 μm in Ven/HNG40; *P* < .001 vs Ven). The hypertrophic cell size was also smaller in venetoclax-treated bones (see Fig. 2C; 10.6 ± 0.2 μm vs 16.3 ± 0.6 μm in control; *P* < .001) and again, this effect was prevented when co-treating with Ven + HNG (see Fig. 2C; 14.5 ± 0.6 μm in Ven/HNG10 and 17.3 ± 0.5 μm in Ven/HNG40; both *P* < .001 vs Ven). Mechanistic studies showed that venetoclax reduced the anti-apoptotic protein BCL-2 and the proliferative marker PCNA while increasing the pro-apoptotic protein Bax (Fig. 3A and 3B). Cotreatment with HNG significantly suppressed the proapoptotic Bax expression, while for PCNA and BCL2 no significant rescuing effect was detected (see Fig. 3A and 3B).

### *In Vivo* Bone Effects of Venetoclax and HNG in Neuroblastoma Mice

To explore the systemic effect of venetoclax and HNG treatment in an experimental *in vivo* model of cancer in which venetoclax is currently being evaluated in clinical trials, a neuroblastoma xenograft mouse model was established. In this 14-day-treatment study in 5-week-old female mice engrafted with neuroblastoma, venetoclax (100 mg/kg/day) was confirmed to suppress tibia bone growth, while HNG at a single dose tested (100 μg/kg/day) did not significantly rescue the growth compared to venetoclax alone, but growth rate was not significantly different compared to the control group (Table 1). Histomorphometric analyses showed that venetoclax reduced the tibia growth plate height, and this was not affected by the co-treatment with HNG (Supplementary Fig. 1 [33]). Venetoclax, venetoclax + HNG, and HNG alone did not affect any of the bone parameters studied when compared to control (Supplementary Table 1; pQCT scans; total bone mineral density; trabecular and cortical bone parameters [33]). The effects of the different treatments on tumor growth are shown in Supplementary Fig. 2 [33].

**Table 1.** Summary of growth data from neuroblastoma-bearing mice treated with venetoclax and HNG

Parameter	Control	Ven	Ven/HNG	HNG
Body mass at end point, g	27.7 ± 2.2	27.5 ± 2.9	27.4 ± 0.9	27.8 ± 2.0
Growth rate, μm/d	39.2 ± 11.5	30.2 ± 9.9*^a^*	34.9 ± 8.8	34.8 ± 9.9
Growth plate height, μm	150.3 ± 9.8	138.9 ± 9.0*^b^*	138.3 ± 10.0*^b^*	149.1 ± 14.9

Histomorphometric analyses were performed in tibia growth plates collected at the end of the experiment (day 14) and 24 hours after the last treatment. Data are shown as means ± SDs, n = 12 to 14/group.

Abbreviation: Ven, venetoclax.

^*a*^ *P* less than .05 vs control. *^b^P* less than .01 vs control.

### Local Effects of Venetoclax and HNG in Cultured Human Growth Plate Cartilage and Human Chondrocytes

**Figure 4.**
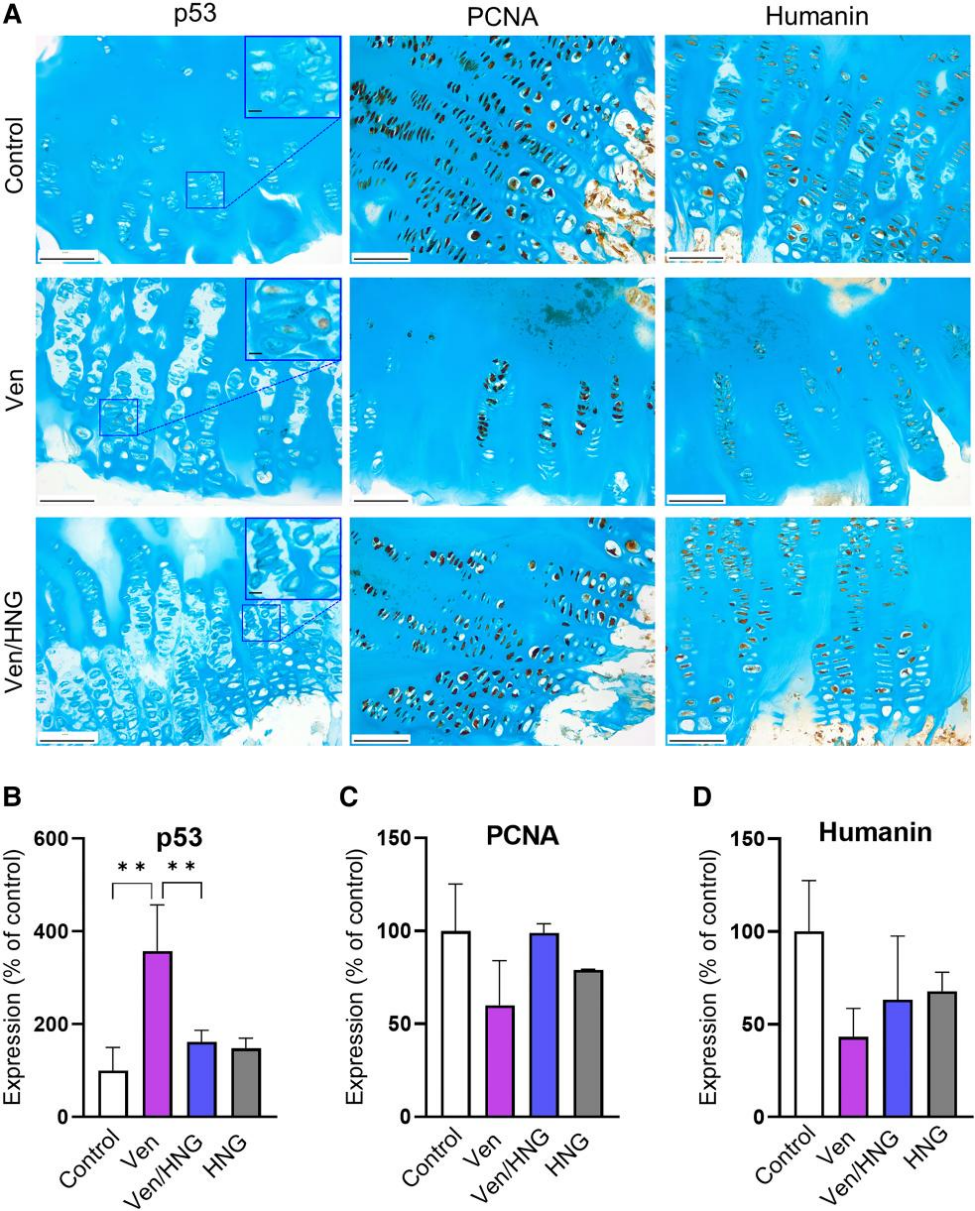
Effects of venetoclax (Ven) and/or HNG treatment on protein expression in *ex vivo* cultured human growth plate tissue. A, Representative images of human growth plate tissues treated with Ven, HNG, or the combination of Ven/HNG for 48 hours and analyzed by immunohistochemistry. Alcian blue was used as counterstain. Original magnification 20×, scale bar = 100 μm. B-D, Quantification of p53, PCNA, and humanin protein expression. Individual cultures of 3 different pieces of growth plate tissue were analyzed. Data are shown as means ± SDs, n = 3/group; **P less than .01.

In an attempt to verify our animal data in human tissues, multiple pieces of growth plate cartilage were microdissected out from growth plate biopsies obtained from one patient. The growth plate specimens were divided into 4 groups and were treated with venetoclax (5 μμ), HNG (40 μμ), the combination of venetoclax and HNG, or vehicle (control group). Venetoclax significantly upregulated the expression of p53, while when co-treating with HNG this was prevented (Fig. 4; 357.5 ± 99.7% of control in Ven; *P* < .01 vs control; 161.6 ± 25.1% of control in Ven/HNG; *P* < .01 vs Ven). The expression levels of PCNA and endogenous humanin did not significantly differ between venetoclax, venetoclax + HNG, and control (*P* = .08 and *P* = .1 for PCNA and humanin, respectively, see Fig. 4). When analyzing gene expression in human chondrocytes, the combination of venetoclax/HNG significantly upregulated the expression of *STAT3* and the autophagy marker *ATG7* compared to venetoclax monotherapy but no significant differences were detected in *SOX9* (Supplementary Fig. 3 [33]).

## Discussion

In the present study, venetoclax was found to suppress longitudinal bone growth when administered locally to *ex vivo* cultured rat metatarsal bones and systemically to neuroblastoma mice. The humanin analog HNG efficiently prevented venetoclax-induced growth impairment in *ex vivo* cultured metatarsals, restoring the growth plate microstructure. Mechanistic studies showed that HNG treatment prevented venetoclax-induced expression of the pro-apoptotic proteins Bax and p53 in rat metatarsals and human growth plate tissues, respectively.

The culture of embryonic murine metatarsal bones is a well-established experimental model enabling real-time studies of longitudinal bone growth in a controlled local milieu where growth plate chondrocytes in different stages of proliferation/differentiation can be monitored [34]. By applying this *ex vivo* model, we could explore the local effects of treatment with venetoclax and/or HNG. Of note, we observed that co-treatment with HNG not only prevented venetoclax-induced growth impairment but also restored the histomorphology and the organization of the growth plate chondrocytes. Interestingly, in a similar organ culture system we earlier reported that shockwave treatment can prevent growth retardation and growth plate disorganization caused by vismodegib, an Indian hedgehog inhibitor [29].

Humanin and its synthetic analogs such as HNG belong to the family of mitochondrial-derived peptides that have gained considerable interest due to their regulatory role in various biological processes including metabolism, inflammation, and oxidative stress [19]. Interestingly, humanin has been shown to suppress the apoptotic cascade via directly binding to Bax and preventing cytochrome c release from the mitochondria [35, 36]. The anti-apoptotic effects were demonstrated in preclinical studies where exogenously administered humanin/HNG counteracted apoptosis-related cell damage caused by chemotherapeutic drugs when studied in male germ cells, leukocytes [37, 38], and growth plate chondrocytes [10, 17]. Of note, our present data are consistent with these findings as we observed that the addition of HNG to venetoclax resulted in a significant downregulation of Bax expression in the metatarsal growth plate.

We also investigated the p53 protein and report that venetoclax treatment significantly upregulated the expression when studied in cultured human growth plate tissue samples. p53 acts as a tumor suppressor that has also been linked to a plethora of biological processes, including apoptosis, autophagy, cell cycle, and DNA damage response [39], while its role in bone and cartilage has not been fully elucidated. Previous studies have shown that p53 negatively regulated osteoblastogenesis [40] and that proteasome inhibition upregulated p53 expression in murine and human growth plate chondrocytes, thus illustrating a mechanistic link between permanent growth suppression and aberrant activation of the apoptotic pathway [41]. Our analyses showed that when HNG was added in combination with venetoclax, the p53 levels were significantly suppressed and this observation provides a novel perspective regarding the cytoprotective actions of humanin/HNG in growth plate chondrocytes.

In the context of longitudinal bone growth, previous studies from our group have demonstrated that HNG has the potential to prevent growth retardation caused by proteasome inhibitors or glucocorticoids when studied in tumor-bearing mouse models and healthy mice, respectively [16, 17]. In the present study, we did not observe a significant bone growth–rescuing effect when comparing the combination treatment of venetoclax and HNG to venetoclax monotherapy in the xenograft neuroblastoma mouse model. However, the present study's short duration, including a treatment window restricted to 14 days due to the rapid tumor growth in the control group, and testing only one dose of HNG might have underestimated the potential for HNG to carry on having bone growth–rescuing effects if studied over a longer time in this disease model. In addition, there is some indication that HNG partially exerted protective effects as the growth rate in the venetoclax/HNG group did not differ significantly from the control group. Regarding tumor progression, HNG neither showed any significant tumor suppression in our neuroblastoma mouse model, nor did it promote tumor growth. These results are in agreement with our previously reported finding that HNG does not interfere with the anticancer activity of proteasome inhibitors in neuroblastoma and medulloblastoma xenograft mouse models [16]. Nevertheless, these findings should be interpreted with caution but remain encouraging and suggest that HNG warrants further investigation in the cancer setting.

It is important to recognize that the growth-rescuing effects of HNG were observed in *ex vivo* models where dose-response studies could be performed in a controlled milieu. The *in vivo* study could not verify a growth-rescuing effect of HNG, and this may be linked to several limitations in our *in vivo* study. First, only one dose of HNG was tested. Second, the duration of treatment was limited to 2 weeks due to rapid tumor growth in the control group, which is a relatively short window to observe any growth-rescuing effects. Third, in the murine model used, the growth plates do not normally fuse as they do in humans. Fourth, only female neuroblastoma mice were studied. Although we did not identify any significant antitumor effects of venetoclax, our results agree with previous studies reporting limited efficacy of only 2 weeks’ monotherapy with venetoclax [42-44]. Finally, due to very limited access to human growth plate cartilage, we were able to study growth plate samples derived from only one patient.

To summarize, our results indicate that the humanin analog HNG can locally prevent venetoclax-induced suppression of longitudinal bone growth and restore growth plate microstructure in cultured metatarsal bones. Our promising *ex vivo* data should be regarded as proof of concept, providing a rationale for further preclinical investigations including different *in vivo* cancer models aiming at future clinical implementation optimizing improved anticancer therapy with limited chronic health conditions.

## Data Availability

Some data sets generated during and/or analyzed during the current study are not publicly available but are available from the corresponding author upon reasonable request.

## Abbreviations

PCNA: proliferating cell nuclear antigen
pQCT: peripheral quantitative computed tomography
R + P: resting + proliferative
Ven: venetoclax
